# Psychosocial Risk and Recurrent Hospitalizations in Women and Men Following LVAD Implantation: A Multi-State Analysis of the INTERMACS Registry

**DOI:** 10.3390/jcdd12060198

**Published:** 2025-05-22

**Authors:** Lisa-Marie Maukel, Sandra Schmeller, Gerdi Weidner, Jan Beyersmann, Heike Spaderna

**Affiliations:** 1 Department of Health Psychology, Trier University, 54296 Trier, Germany; maukel@uni-trier.de; 2 Institute of Statistics, Ulm University, 89081 Ulm, Germany; sandra.schmeller@uni-ulm.de (S.S.); jan.beyersmann@uni-ulm.de (J.B.); 3 Department of Biology, San Francisco State University, San Francisco, CA 94132, USA; gweidner@sfsu.edu

**Keywords:** heart failure, clinical outcomes, rehospitalization, women, LVAD, psychosocial risk

## Abstract

Background: Women experience higher rates of adverse events and first rehospitalization after left ventricular assist device (LVAD) implantation compared with men. This study investigated the role of sex and preimplant psychosocial risk in recurrent hospitalizations. Methods: Data from 20,123 INTERMACS patients (21.3% women) were analyzed. Cumulative transition rates (e.g., home to hospitalization) were estimated and Andersen–Gill models, adjusted for covariates, examined the association between sex, preimplant psychosocial risk, and cumulative transition hazards for rehospitalization. State occupation probabilities, the mean number of hospitalizations, and the cumulative average length of hospital stay were calculated and stratified by sex and psychosocial risk. Results: Psychosocial risk preimplant was more prevalent in men than in women (21.4% vs. 17.5%, *p* < 0.001). The interaction of female sex and psychosocial risk increased rehospitalization hazards, independently of covariates [HR_adj_ 1.11, 95% CI (1.01–1.22), *p* = 0.036]. One-year postimplant, women with vs. without psychosocial risk had 2.2 vs. 1.8 hospitalizations, while men experienced 1.8 vs. 1.7 hospitalizations, respectively. Women with vs. without psychosocial risk spent 20 vs.16 days hospitalized, and men 15 vs. 14 days (all *p* < 0.001). Conclusions: Preimplant psychosocial risk independently contributed to recurrent hospitalizations post-LVAD, particularly affecting women. The early identification and management of these factors may reduce rehospitalizations and improve clinical outcomes.

## 1. Introduction

Left ventricular assist devices (LVADs) have become a standard treatment for individuals with heart failure and reduced ejection fraction (HFrEF), with reported survival rates of approximately 80% at 1 year and 40–50% at 5 years [1,2]. However, outcomes and utilization differ markedly by sex. Women represent a larger proportion of the overall heart failure (HF) population and have a higher incidence of HF than men [3]. While most women are diagnosed with HF with preserved ejection fraction (HFpEF), they still comprise approximately 40% of the HFrEF population [4]. Despite this, women remain significantly underrepresented in advanced HF therapies: compared to men, women are 72% less likely to undergo heart transplantation and 62% less likely to receive an LVAD [5]. Consequently, women account for only about 20% of LVAD recipients [6].

Historically, women had higher mortality than men after LVAD implantation during the era of pulsatile-flow devices [7]. However, the transition to newer-generation continuous flow (CF)-LVADs—some with improved size and flow characteristics better suited to female physiology—has been associated with improved outcomes in women [7,8,9].

Despite these advances, sex differences persist. Women continue to experience higher rates of adverse events such as bleeding and infections compared to men [10,11,12,13] and are also more likely to experience their first rehospitalization within 1-year postimplant (72.1% vs. 68.9%) [11].

Psychosocial factors, such as substance abuse and psychiatric diagnoses, have been identified as significant determinants of postimplant outcomes in both sexes [14,15,16]. Particularly in women, the presence of preimplant psychosocial risk was associated with a 15% increase in first hospitalization rates, compared to 3% in men. This association was independent of device-related (e.g., axial vs. centrifugal flow) and clinical risk factors (e.g., primary diagnoses, differences in medication management) [11].

Notably, these findings only account for patients’ first hospitalization after LVAD implantation. However, patients on average experience two hospitalizations only within the first year after implant [8,17], with longer follow-up data rarely reported. These recurrent hospitalizations are associated with increased patient burden, higher mortality risk, and rising healthcare costs [1,18].

Findings regarding the role of female sex for recurrent hospitalizations are inconclusive. While women were found to have a higher risk of 30-day readmissions compared with men [19,20], no significant sex differences were observed in days alive and out of hospital [21] or length-of-stay [22]. These studies, however, differed in follow-up durations and applied statistical approaches that, unlike multi-state modeling, did not adequately account for recurrent events. Moreover, these studies did not consider preimplant psychosocial risk factors.

This study aims to address these gaps using a multi-state analysis of recurrent hospitalizations in a large dataset from the Interagency Registry for Mechanically Assisted Circulatory Support (INTERMACS) registry. The objectives are to (1) describe sex-specific patterns in cumulative transition rates (e.g., home to hospital) and to (2) analyze the interaction of preimplant psychosocial risk and sex on cumulative transition hazards from home to hospital while adjusting for clinical, demographic, and behavioral covariates. We hypothesize that women will experience higher transition rates from home to hospital compared to men, particularly due to the impact of preimplant psychosocial risk. In addition, we explore the probability for being in a state of “hospitalization” throughout the follow-up, the mean number of hospitalizations, and cumulative average lengths of hospital stay at each possible time point of the observation, stratified by sex and preimplant psychosocial risk.

## 2. Material and Methods

### 2.1. Study Population

INTERMACS is a North American prospective registry that enrolls patients with advanced heart failure undergoing durable mechanical circulatory support. Prior to implantation, the registry records clinical, demographic, behavioral, and psychosocial patient characteristics. Patients are monitored postimplantation for adverse events and rehospitalizations until they experience death, undergo heart transplantation, or recover [23]. This INTERMACS analysis included de-identified data from adult patients aged over 18 years at the time of implantation. Informed consent was obtained from all participants before implantation by the participating centers. Patients who received pulsatile-flow LVAD, right ventricular assist device, biventricular assist device, or total artificial hearts were excluded. Data from 20,123 patients (21.3% women), registered between July 2006 and December 2017, with primary CF-LVAD were analyzed. Data were accessed through the Biological Specimen and Data Repository Information Coordinating Center (BioLINCC). This study received approval from the Institutional Review Board at Trier University (amendment 26 April 2024 to EK no. 66/2018).

#### Preimplant Characteristics and Psychosocial Risk

In addition to clinical factors [10,14], demographic and behavioral variables such as age, race, employment status, marital status, educational attainment, BMI, and smoking status were included in the analysis. The psychosocial variables of INTERMACS’ *concerns and contraindications for transplant* were limited social support, limited cognition/understanding, alcohol abuse, drug abuse, severe depression, other major psychiatric diagnosis, and repeated noncompliance, covering all five key psychosocial domains as suggested by the Consensus Statement on psychosocial evaluation [24,25]. Based on previous approaches [11,14], a binary psychosocial risk variable was constructed (present = 1, not present = 0). Psychosocial risk was coded “present” if at least one of the above conditions was identified in a patient preimplant.

### 2.2. Outcome Measurements

In recurrent time-to-event analysis, some states, such as hospitalizations, can occur multiple times and are referred to as ‘recurrent’ events. Other states, such as death, are considered absorbing because, once patients transition to an absorbing state, they are no longer at risk for subsequent events. This competing risk framework within the recurrent event analysis ensures that changes in risk sets are appropriately accounted for. Considering all observed events, including all recurrent hospitalizations and absorbing states—rather than focusing solely on the first event per patient, as is common in traditional time-to-event analyses—provides a more comprehensive understanding of disease progression and enhances statistical power [26,27].

**Figure 1:**
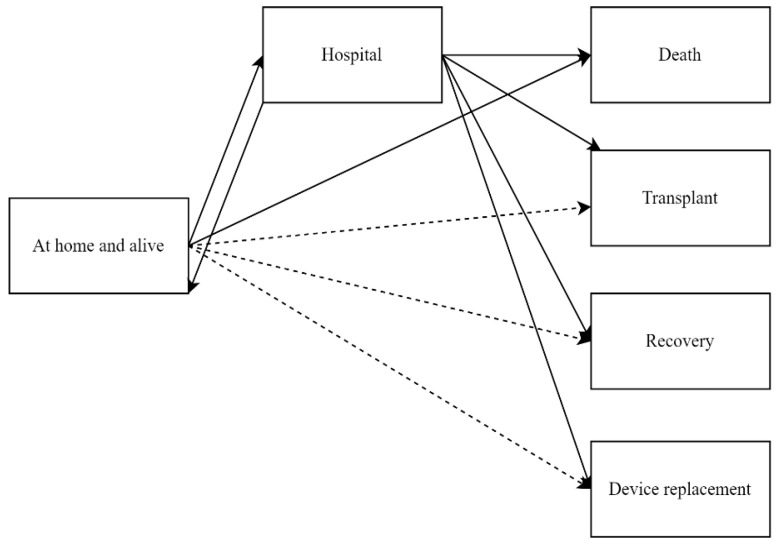
Multi-state model. The states are depicted as boxes, and the possible transitions between states are indicated by arrows. Dashed arrows represent transitions that are included in the model but are not discussed further, as they are clinically irrelevant and would typically involve scheduled hospital appointments rather than unscheduled rehospitalizations.

In our multi-state model (Figure 1), we defined ‘alive and at home’ and ‘hospitalized’ as the recurrent states of interest. Thus, our outcomes of interest include all-cause recurrent hospitalizations for each patient, as long as they remained under observation. Each event “rehospitalization” represents a change in status from being ‘alive and at home’ to being ‘hospitalized’, followed by a return to the ‘alive and at home’ state. The model also accounts for the transitions from hospital to the absorbing states death, heart transplantation, device replacement due to complications, device explantation due to recovery, and from home to death. Time-to-event was calculated as the time from CF-LVAD implantation until one of these outcomes occurred, accounting for recurrent states, until the end of follow-up.

### 2.3. Statistical Analysis

We applied the semiparametric imputation procedure [28] to handle missing data in all covariates, including psychosocial risk, as recommended when missing data are less than 30%. Sensitivity analyses were conducted using complete case models, which showed consistent results compared to the single-imputed model.

Continuous variables were summarized using means and standard deviations; categorical variables were described with frequencies and percentages. Sex differences in preimplant characteristics were assessed using *t*-tests for continuous variables and chi-square tests for categorical variables.

Recurrent hospitalizations were analyzed by applying multi-state models. Cumulative partly conditional transition rates, calculated under the condition of being at risk for the event shortly before time *t*, were estimated for each transition in Figure 1 using the nonparametric Nelson–Aalen estimator in non-Markov models [29,30].

To estimate the effects of covariates on the transition hazard from home to hospitalizations, we utilized the semiparametric Andersen–Gill model. To assess the Markov assumption—which posits that the occurrence of the next event is independent of previous events given the current state and time—we included the number of previous events as a covariate. After having observed a violation of this assumption, we applied the proportional rate model proposed by Gosh and Lin [27] and compared its results from univariable analyses with those of the Andersen–Gill model. As the results were similar, and the Gosh and Lin model required excessive computational resources, we proceeded with the Andersen–Gill model for multiple analyses. Results are reported as hazard ratios (HRs) with corresponding 95% confidence intervals (CIs). The multivariable regression model for transition hazards from home to hospitalizations included the following variables: sex, psychosocial risk (yes vs. no), interaction between sex and psychosocial risk, age, race, working for income, marital status, education, BMI, and smoking status. Clinical covariates were device strategy, primary diagnosis, time since diagnosis, previous cardiac surgeries, left ventricular end-diastolic diameter (LVEDD), INTERMACS profile, pump type (axial vs. centrifugal), implantable cardioverter–defibrillator (ICD), pulmonary hypertension, albumin, bilirubin, creatinine, BUN, platelet count, severe diabetes, and medications (Beta-Blockers, ACE, ARB, Aldosterone).

To provide probabilities for recurrent hospitalizations in addition to cumulative transition rates and hazards, we estimated marginal probabilities based on the Aalen–Johansen estimator, which can be utilized even when the Markov assumption is not met (see above) [30,31]. These estimates include state occupation probabilities, which represent the likelihood of a patient being in a particular state, such as being hospitalized or in any absorbing state, at a given time. Based on these probabilities, the mean number of hospitalizations and the cumulative average length of hospital stay were calculated and stratified by sex and psychosocial risk (yes vs. no).

Statistical significance was set at *p* < 0.05. All analyses were performed using R, version 4.3.3, including the packages mice, mvna, survival, etm, and reReg [32].

## 3. Results

### 3.1. Sex Differences Preimplant

Of the 20,123 patients receiving a primary CF-LVAD, 4282 (21.3%) were female. There were differences between women and men preimplant (Table 1). Women were less likely to have psychosocial risk than men (17.5% vs. 21.4%, *p* < 0.001) [11]. Specifically, women were less likely than men to have a history of substance use (tobacco, alcohol, drugs), but they were more likely to be current smokers and more often depressed. However, there were no sex differences in limited social support and limited cognition/understanding [11].

### 3.2. Sex Differences in State Transitions

**Figure 2:**
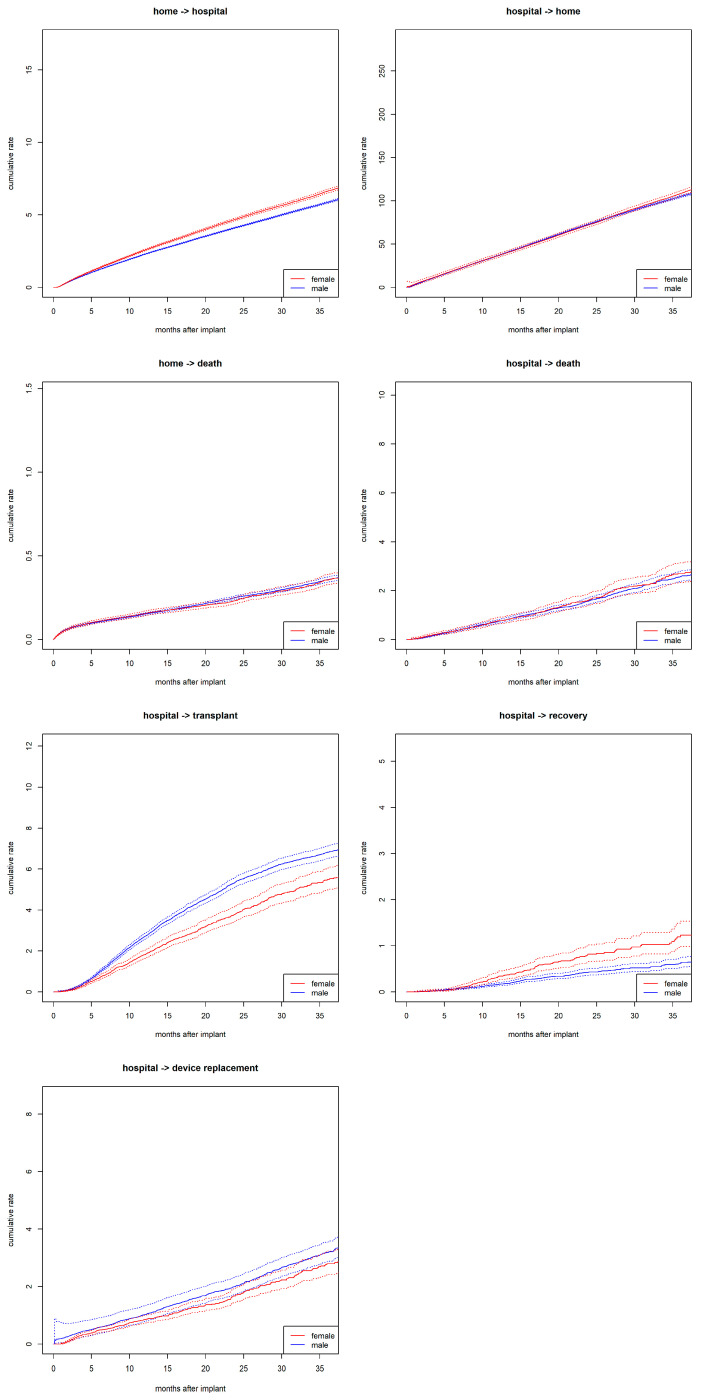
Transition rates. Transition rates between states (e.g., “home” to “hospital”) stratified by sex and calculated as Nelson–Aalen estimator with 95% CI (dashed lines).

During the observation period, over 135,000 transitions were recorded. Among these, 60,869 were rehospitalizations—transitions from home to hospital. This included 14,018 rehospitalizations in women and 46,851 in men. Women had increased rates for transitioning from home to hospital compared to men (Figure 2). Additionally, women had higher rates for device explant due to recovery compared to men but lower rates for undergoing transplantation while hospitalized. No sex differences were observed in the transition rates from home to death or from hospital to death. Independent of sex, discharge from the hospital is the most likely, while recovery within the hospital is the least likely of all transitions.

In the multiple regression models, female sex [HR_adj_ 1.11, 95% CI (1.06–1.16), *p* < 0.001], psychosocial risk [HR_adj_ 1.07, 95% CI (1.02–1.12), *p* = 0.003], and the interaction of both [HR_adj_ 1.11, 95% CI (1.01–1.22), *p* = 0.036] were significantly associated with increased transition hazards for home to hospital, even after controlling for clinical, demographic, and behavioral covariates (Table 2).

Demographic and behavioral risk factors associated with increased transition hazards for home to hospital were not working for income [HR_adj_ 1.05, 95% CI (1.01–1.10), *p* = 0.021], obesity [HR_adj_ 1.09, 95% CI (1.05–1.13), *p* < 0.001], currently smoking [HR_adj_ 1.10, 95% CI (1.02–1.18), *p* = 0.013], and smoking history [HR_adj_ 1.07, 95% CI (1.03–1.11), *p* < 0.001] (Table 2). Higher educational attainment was associated with decreased transition hazards from home to hospital [HR_adj_ 0.92, 95% CI (0.87–0.97), *p* = 0.004].

### 3.3. Probability of Being Hospitalized

The state occupation probability for being in hospital decreased during follow-up and the probability of being hospitalized was higher for women than for men during the first 25 months postimplant. There were no sex differences in the state occupation probability of being in any absorbing state (see Figures S1 and S2).

### 3.4. Mean Number of Hospitalizations

**Figure 3:**
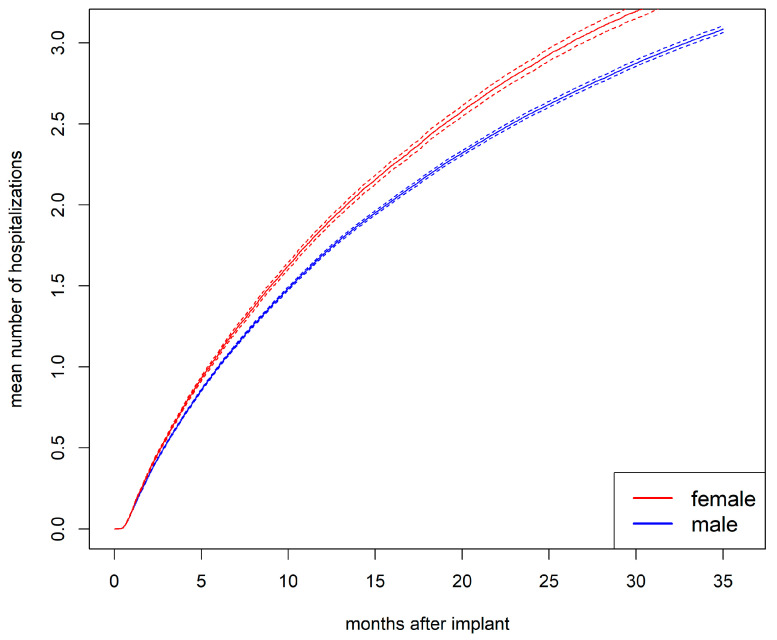
Mean number of hospitalizations with bootstrapped 95% CI (dashed lines) stratified by sex.

The mean number of hospitalizations was higher for women than for men (Figure 3). This difference cannot be explained by men entering an absorbing state earlier and thus avoiding rehospitalizations, as no sex differences were observed in the state occupation probability of being in an absorbing state (see Figure S2). After 1 year, the mean number of hospitalizations was 1.9 for women and 1.7 for men (*p* < 0.001). After 2 years, it was 2.9 for women and 2.6 for men (*p* < 0.001).

**Figure 4:**
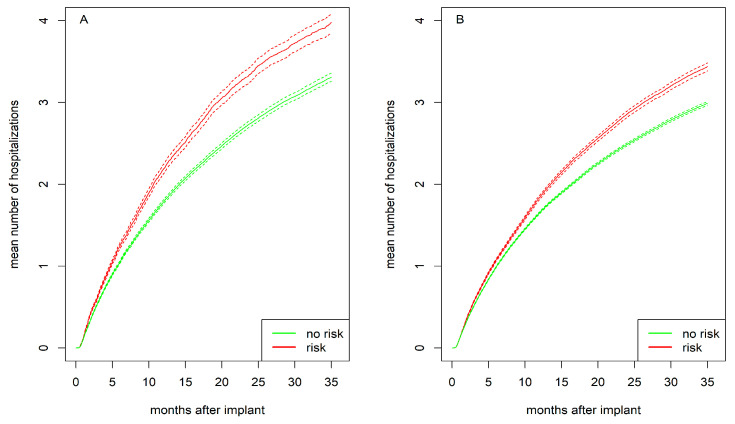
Mean number of hospitalizations with bootstrapped 95% CI (dashed lines) for (**A**) women and (**B**) men stratified by psychosocial risk (yes vs. no).

Figure 4 presents the mean number of hospitalizations for women and men separately, stratified by psychosocial risk. Individuals with psychosocial risk experienced more hospitalizations compared to those without such risk, with this difference being more pronounced in women than in men. After 1 year, women with psychosocial risk had on average 2.2 hospitalizations compared to 1.8 for women without risk (*p* < 0.001). Men with psychosocial risk had 1.8 hospitalizations compared to 1.7 for men without risk (*p* < 0.001). After 2 years, women with psychosocial risk had 3.4 hospitalizations compared to 2.8 for women without risk (*p* < 0.001). Men with psychosocial risk had 2.9 hospitalizations compared to 2.5 for men without risk (*p* < 0.001).

### 3.5. Cumulative Average Length of Stay in Hospital

**Figure 5:**
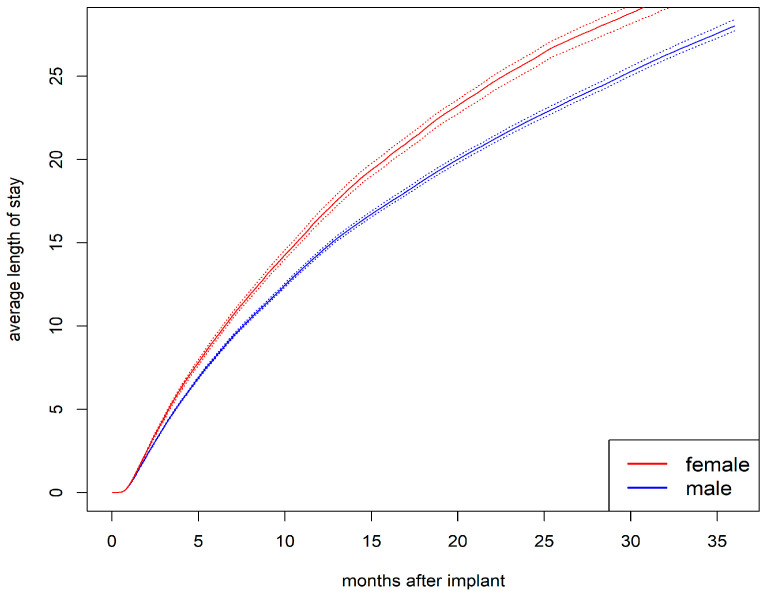
Average length of stay in the hospital with bootstrapped 95% CI (dashed lines) stratified by sex.

The cumulative average length of hospital stay was longer for women than for men (Figure 5). After one year, the average length of stay was 16.5 days for women compared to 14.4 days for men (*p* < 0.001). After two years, it was 25.8 days for women compared to 22.3 days for men (*p* < 0.001).

**Figure 6:**
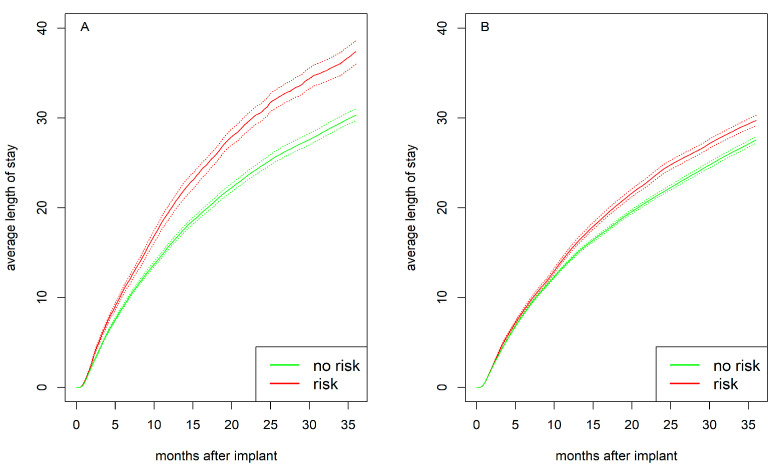
Average length of stay with bootstrapped 95% CI (dashed lines) for (**A**) women and (**B**) men stratified by psychosocial risk (yes vs. no).

Psychosocial risk preimplant was associated with a longer average hospital stay, with the difference being more pronounced in women (Figure 6). This suggests that the sex difference observed in Figure 5 is driven by the interaction with psychosocial risk. Notably, after one year, women without psychosocial risk (15.8 days) and men without psychosocial risk (14.2 days) had similarly long hospital stays, whereas women with psychosocial risk had markedly longer stays than women without psychosocial risk (19.7 days; *p* < 0.001). Men with psychosocial risk also had longer stays than men without psychosocial risk (15.2 days; *p* < 0.001), but the difference was less pronounced. After two years, women with psychosocial risk had an average hospital stay of 30.8 days compared to 24.8 days for women without risk (*p* < 0.001). Men with psychosocial risk had 24.3 days compared to 21.7 days for men without risk (*p* < 0.001).

## 4. Discussion

In multi-state recurrent event analysis with data from over 20,000 patients, women with CF-LVADs experienced higher rates of recurrent hospitalizations compared to men, particularly in the presence of psychosocial risk. This pattern remained significant even after adjusting for clinical, demographic, and behavioral variables. This extends our previous finding that psychosocial risk at time of LVAD implantation was associated with a 15% increased rate of first hospitalization among women, compared to a 3% increased rate in men [11]. Importantly, the present findings show that the influence of preimplant psychosocial risk in women appears to persist beyond the first hospitalization, with the interaction between psychosocial risk and female sex increasing the risk of recurrent hospitalizations by 11%. The application of a multi-state model over a 36-month observation period addresses methodological challenges in analyzing sex differences in recurrent hospitalization [19,20,21,22] and thereby contributes to a more detailed clinical picture of outcomes [26,27].

While previous studies reported an average of two hospitalizations per year per patient [8,17], our analysis provides a more nuanced perspective by incorporating longer follow-up and subgroup analyses. After one year, women with psychosocial risk had a mean of 2.19 hospitalizations, compared to 1.78 for those without risk. Men with psychosocial risk averaged 1.84 hospitalizations, compared to 1.65 for their counterparts without risk. After two years, women with psychosocial risk experienced nearly one more hospitalization on average than men without psychosocial risk. These findings highlight that the long-term hospitalization burden is highest among women with psychosocial risk.

In women, psychosocial risk preimplant was not only associated with an increased rate of rehospitalizations, but also with longer hospital stays compared to both women without such risks and men, regardless of their psychosocial risk status. After one year, women with psychosocial risk spend an average of 20 days in the hospital, compared to 16 days for women without psychosocial risk. In men, psychosocial risk made only a difference of 1 day (15 days vs. 14 days). Psychosocial factors appear to have a particularly strong impact on women’s hospital stay duration.

Overall, psychosocial risk factors preimplant were associated with increased rates for rehospitalizations and accordingly, with a higher mean number of hospitalizations, particularly in women. In addition, women with psychosocial risk had longer hospital stays. Thus, psychosocial risk appears to contribute directly to their greater burden and increased healthcare costs [1,18]. Still, it is unclear whether patients with psychosocial issues receive psychological help during their hospitalization.

Distinguishing between biological sex and social gender is essential for epidemiological research [33,34]. Biological sex refers to inherent physiological characteristics such as sex chromosomes, hormones, and reproductive organs, whereas gender encompasses gender identity, socially constructed roles and responsibilities, institutionalized gender norms, and power relations associated with being perceived as a woman or a man [33,35]. In cardiovascular disease outcomes, sex and gender have been shown to exert distinct—and at times divergent—influences [36]. For example, female sex has been associated with a 50% reduction in the risk of major adverse events in young adults with acute coronary syndrome, potentially attributable to the protective effects of estrogen. In contrast, gender-related characteristics traditionally ascribed to women—such as responsibilities for household chores, low personal income, and personality traits like yielding and sensitivity to others’ needs—have been associated with a fivefold increase in the risk of major cardiac events [37]. In this LVAD cohort, the average age of women is 54 years. The protective effects of female sex hormones may be diminished at this age, which allows us to draw conclusions with regard to gender-related risk factors. Factors, such as perceived social support and gender-specific family roles, not included in the INTERMACS, likely influence women’s healthcare experiences and health behaviors. For example, cardiologists have noted that male patients at heart transplant centers are typically accompanied by their wives, whereas female patients are seldom accompanied by their husbands [38]. Moreover, men with heart failure tend to name their spouse as the primary caregiver, whereas women more often identify parents or adult children [39]. There is also emerging evidence that clinicians perceive male caregivers as less effective in the context of heart failure management [40]. A recent study examining dyadic self-management in heart failure identified two predominant patterns: collaborative and autonomous. The autonomous pattern, characterized by less shared responsibility between partner, was more commonly observed among dyads comprising female patients and male caregivers. Patients in this group exhibited higher levels of anxiety and depression, and reported lower relationship quality compared to those in collaborative dyads [41]. This is of particular concern given that marital stress is a known predictor of adverse cardiovascular outcomes, especially among women [42]. These findings may reflect entrenched gender norms wherein women are expected to assume caregiving roles, potentially leaving them with insufficient support when they themselves become patients. In addition, structural barriers—such as limited access to flexible appointment scheduling, transportation, and caregiving relief—disproportionately burden women [43,44]. These factors may create competing demands between healthcare engagement and caregiving duties, potentially leading to suboptimal self-care, delayed care-seeking, and prolonged or more frequent rehospitalizations [44]. Future research to explore factors contributing to the association between psychosocial risk and disparities in LVAD outcomes for women and men is clearly warranted and needs to consider both biological factors and gender-related factors [35,40,43].

Independent of gender and sex, this research also suggests that behavioral risk factors such as obesity and smoking play a role for clinical outcomes after LVAD implantation [11,45,46]. For example, current smoking was associated with a 10% increase in rehospitalization rates. Additionally, lower education levels and not working for income were linked to higher rehospitalization rates. These socioeconomic risk factors can limit healthcare access and delay the seeking of medical attention [47], thereby exacerbating health issues that may lead to hospitalization. Particularly, the interplay of psychosocial, behavioral, and socioeconomic risk factors presents significant challenges for LVAD patients, highlighting the need for comprehensive support and intervention strategies

It is intriguing that, despite sex differences in rehospitalizations, this multi-state analysis replicated comparable survival rates for women and men on LVAD [7,9,13]. Additionally, it supports earlier findings suggesting that women may be more likely to experience cardiac recovery [48]. To further improve outcomes for women and reduce rehospitalizations, the close monitoring of adverse events and complications is essential. The timely detection of psychological comorbidities, such as anxiety and depression, are crucial, and referring patients to psychological care promptly ensures that they receive appropriate support [49,50].

This is particularly important since previous research has shown that women with LVAD are more likely to have depression and other major psychiatric diagnoses, while men are more likely to have substance use issues [11]. This suggests the need for a gender-sensitive approach to address these psychosocial issues in targeted prevention and intervention strategies for patients with LVAD. Psychosocial interventions have already been shown to improve the quality of life, decreasing symptoms of depression and anxiety in patients with heart failure [51,52]. When tailored to individual needs and gender-specific factors, these interventions not only improve psychological well-being but may also lead to better clinical outcomes [53].

### Limitations

The assessment of psychosocial variables in INTERMACS—recorded under *concerns and contraindications for transplant*—may vary across participating centers. While a substantial proportion of sites reportedly use validated tools such as the Stanford Integrated Psychosocial Assessment for Transplant (SIPAT), there is likely variation in both the mode of assessment and adherence to standardized protocols [54]. This heterogeneity may introduce measurement bias, particularly in multicenter analyses. The implementation of standardized, psychometrically robust tools that are capable of capturing psychosocial characteristics consistently and longitudinally would enhance the reliability of models in evaluating the impact of psychosocial risk on LVAD outcomes. Moreover, such tools would facilitate the assessment of changes in psychosocial risk over time, including post-rehospitalization, thereby informing timely interventions and optimizing patient care. Nevertheless, INTERMACS includes data across five core psychosocial domains—social support, cognition, substance use, psychopathology, and nonadherence [24,25]—providing a valuable basis for capturing patients’ overall psychosocial risk profiles.

This study examined the relationship between sex, psychosocial risk, and recurrent hospitalizations for all causes, a commonly used outcome in large epidemiological studies [55]. A more granular analysis of specific reasons for rehospitalization could provide valuable insights for clinicians, helping to refine targeted interventions. Unfortunately, the predominant reason for rehospitalization in both women (19.0%) and men (17.0%) was categorized as ‘Other’ in the INTERMACS registry (Table S1), which limits the ability to further differentiate and explore the specific causes of these readmissions. While we acknowledge that this limits the ability to draw clinical conclusions about how sex and psychosocial risk factors affect specific causes of rehospitalization, we believe that the influence of gender-related structural and relational factors on the likelihood of rehospitalization—independent of the underlying medical issues—remains a critical area of focus.

Fortunately, newer devices, such as the HeartMate III—which is not included in this INTERMACS dataset—offer better fit for the female body and further reduce rehospitalizations among women [56,57]. It would be valuable to explore the role of psychosocial risk factors on outcomes in women with this generation of devices.

## 5. Conclusions

During the course of life after CF-LVAD implantation, women experience higher rates of recurrent hospitalizations compared to men, particularly in the presence of psychosocial risk, even after adjusting for clinical, demographic, and behavioral variables. Women with psychosocial risk also experience a higher average number of hospitalizations and longer hospital stays compared to women without such risks and men. These findings emphasize the need for comprehensive psychosocial assessments and the development of targeted interventions to improve outcomes post LVAD.

## Figures and Tables

**Table 1:** Preimplant psychosocial, demographic, behavioral, and clinical characteristics for women and men with CF-LVAD.

	Women (*n* = 4282) 21.3%	Men (*n* = 15,817) 78.7%	Total (*N* = 20,123)	*p*-Value
**Psychosocial risk, *n* (%)**	554 (17.5)	2490 (21.4)	3044 (15.1)	<0.001
**Demographic and behavioral characteristics**				
Age in years	54.08 (13.44)	57.55 (12.69)	56.81 (12.93)	<0.001
Educational attainment, *n* (%)				0.222
Up to primary	112 (3.5)	434 (3.7)	546 (3.7)	
Secondary	1464 (45.5)	5284 (45.2)	6750 (45.2)	
Post-secondary	901 (28.0)	3111 (26.6)	4013 (26.9)	
Tertiary	743 (23.1)	2868 (24.5)	3611 (24.2)	
Marital status: married, *n* (%)	2189 (52.4)	10,704 (68.8)	12,896 (65.3)	<0.001
Race White, *n* (%)	2430 (56.7)	11042 (69.8)	13474 (67.0)	<0.001
Working for income, *n* (%)	563 (14.5)	2717 (18.9)	3280 (18.0)	<0.001
BMI, *n* (%)				<0.001
Underweight	208 (4.9)	489 (3.1)	697 (3.5)	
Non-obese	2375 (55.9)	9524 (60.7)	11,909 (59.6)	
Obese	1336 (31.4)	4932 (31.4)	6280 (31.4)	
Morbidly obese	332 (7.8)	752 (4.8)	1086 (5.4)	
Smoking history, *n* (%)				<0.001
Currently	162 (5.1)	611 (5.3)	773 (5.2)	
Past	648 (20.5)	3287 (28.3)	3939 (26.6)	
Never	2347 (74.3)	7716 (66.4)	10,082 (68.1)	
**Clinical variables**				
LVEDD	6.51 (1.08)	6.90 (1.12)	6.82 (1.12)	<0.001
LVAD axial, *n* (%)	3245 (75.8)	12,704 (80.3)	15,961 (79.3)	<0.001
Device strategy, *n* (%)				0.001
Destination therapy	1730 (40.4)	6875 (43.5)	8615 (42.8)	
Bridge to transplant	2515 (58.8)	8840 (55.9)	11,369 (56.5)	
Bridge to recovery	23 (0.5)	55 (0.3)	78 (0.4)	
Rescue therapy	12 (0.3)	39 (0.2)	51 (0.3)	
INTERMACS profile, *n* (%)				0.005
1	723 (16.9)	2454 (15.6)	3182 (15.9)	
2	1541 (36.1)	5683 (36.1)	7230 (36.1)	
Primary ischemic diagnosis, *n* (%)	1236 (29.1)	7911 (50.4)	9160 (45.8)	<0.001
Time since diagnosis, *n* (%)				<0.001
<1 month	269 (6.5)	778 (5.1)	1049 (5.4)	
1 month–1 year	530 (12.8)	1559 (10.2)	2089 (10.8)	
1–2 years	379 (9.2)	985 (6.5)	1365 (7.0)	
>2 years	2947 (71.4)	11,921 (78.2)	14,886 (76.8)	
Previous cardiac surgery, *n* (%)	1088 (25.4)	5689 (36.0)	6784 (33.7)	<0.001
Current ICD, *n* (%)	3248 (76.4)	12,717 (80.9)	15,982 (79.9)	<0.001
Severe diabetes, *n* (%)	314 (9.9)	1130 (9.7)	1445 (9.8)	0.742
Pulmonary Hypertension, *n* (%)	655 (20.7)	2616 (22.5)	3275 (22.1)	0.035
**Preoperative blood values**				
Albumin g/dL	3.38 (0.66)	3.40 (0.65)	3.40 (0.65)	0.071
Bilirubin total mg/dL	1.21 (1.61)	1.42 (1.82)	1.38 (1.79)	<0.001
BUN mg/dL	26.07 (16.86)	30.14 (18.27)	29.27 (18.05)	<0.001
Creatinine mg/dL	1.22 (0.65)	1.45 (0.71)	1.40 (0.70)	<0.001
Platelets × 1000/µL	209.32 (87.46)	193.68 (79.19)	197.00 (81.25)	<0.001
**Medication *n* (%)**				
Beta-Blocker	3140 (75.8)	11,989 (78.5)	15,141 (77.9)	<0.001
ACE	1911 (48.0)	7317 (49.9)	9233 (49.4)	0.034
ARB	817 (20.9)	2555 (18.0)	3372 (18.6)	<0.001
Aldosterone	2457 (60.6)	8307 (55.9)	10,775 (56.9)	<0.001
Loop Diuretics	3605 (84.9)	13,492 (86.2)	17,117 (85.9)	0.046

Note: Preimplant characteristics have been previously reported in [11] but are included here for completeness; original unimputed data; unless otherwise indicated, data are presented as mean (standard deviation); psychosocial risk was computed as binary (any vs. none) if at least one psychosocial risk factor (limited social support, limited cognition/understanding, alcohol abuse, drug abuse, severe depression, other major psychiatric diagnosis, and repeated noncompliance) was applicable. BMI, body mass index; LVEDD, left ventricular end-diastolic diameter; ICD, implantable cardioverter–defibrillator; BUN, blood urea nitrogen; ACE, angiotensin-converting enzyme inhibitor; ARB, angiotensin II receptor blocker.

**Table 2:** Multiple regression model for transition hazards from home to hospital.

	HR (95% CI)	*p*-Value
Female sex	1.11 (1.06–1.16)	<0.001
Psychosocial risk	1.07 (1.02–1.12)	0.003
Female sex × psychosocial risk	1.11 (1.01–1.22)	0.036
Age in 10 years	0.97 (0.96–0.99)	0.002
Race White	0.97 (0.94–1.01)	0.124
Marital status		
Married/domestic partners	REF	
Single	1.04 (0.99–1.09)	0.115
Divorced/widowed	1.05 (1.00–1.10)	0.050
Widowed	1.04 (0.95–1.14)	0.363
Educational attainment	0.92 (0.87–0.97)	0.004
Not working for income	1.05 (1.01–1.10)	0.021
BMI		
Non-obese	REF	
Underweight	1.01 (0.92–1.10)	0.828
Obese	1.09 (1.05–1.13)	<0.001
Morbidly obese	1.08 (1.00–1.16)	0.053
Smoking history		
Never	REF	
Past	1.07 (1.03–1.11)	<0.001
Currently	1.10 (1.02–1.18)	0.013
Device strategy		
Destination therapy	REF	
Bridge to transplant	1.00 (0.97–1.04)	0.916
Bridge to recovery	0.92 (0.74–1.14)	0.447
Rescue therapy	0.76 (0.54–1.08)	0.125
LVEDD	0.99 (0.98–1.00)	0.181
LVAD axial	0.87 (0.84–0.91)	<0.001
INTERMACS profile	1.05 (0.98–1.12)	0.169
Primary diagnosis		
Ischemic	REF	
Idiopathic	0.96 (0.92–1.00)	0.069
Other	0.91 (0.87–0.95)	<0.001
Time since diagnosis	1.10 (1.04–1.17)	0.002
Previous cardiac surgery	1.10 (1.05–1.14)	<0.001
Current ICD	1.12 (1.07–1.18)	<0.001
Severe diabetes	1.09 (1.04–1.14)	<0.001
Pulmonary hypertension	0.99 (0.95–1.03)	0.561
Albumin g/dL	1.01 (0.99–1.04)	0.363
Bilirubin total mg/dL	0.97 (0.95–0.98)	<0.001
BUN mg/dL	1.00 (1.00–1.00)	0.558
Creatinine mg/dL	1.06 (1.03–1.09)	<0.001
Platelets × 1000/µL	1.00 (1.00–1.00)	<0.001
Beta blocker	1.01 (0.97–1.05)	0.671
ACE	0.98 (0.95–1.01)	0.247
ARB	0.99 (0.95–1.03)	0.573
Aldosterone	1.01 (0.98–1.04)	0.575

Note. Educational attainment, INTERMACS profile, and time since diagnosis were modeled as ordinal factors, with a linear trend (low to high) tested for their association with the outcome.

## Data Availability

The INTERMACS data were provided by the National Heart, Lung, and Blood Institute Biologic Specimen and Data Repository Information Coordinating Center. Anonymized data and materials have been made publicly available at the Biologic Specimen and Data Repository Information Coordinating Center of the National Heart, Lung, and Blood Institute and access can be requested at https://biolincc.nhlbi.nih.gov/studies/intermacs/.

## Supplementary Materials

The following supporting information can be downloaded at: https://www.mdpi.com/article/10.3390/jcdd12060198/s1, Table S1: Reasons for rehospitalization in women and men with implanted left ventricular assist devices; Figure S1: State occupation probabilities for being hospitalized stratified by sex; Figure S2: State occupation probabilities for being in any absorbing state (i.e., death, heart transplantation, device replacement due to complications, device explantation due to recovery) stratified by sex. In an absorbing state, patients are no longer at risk for subsequent events, such as rehospitalization.

## Abbreviations

ACE	Angiotensin-converting enzyme inhibitor
ARB	Angiotensin II receptor blocker
BMI	Body mass index
BUN	Blood urea nitrogen
CF-LVAD	Continuous-flow left ventricular assist device
HFrEF	Heart failure with reduced ejection fraction
ICD	Implantable cardioverter–defibrillator
INTERMACS	Interagency Registry for Mechanically Assisted Circulatory Support
LVAD	Left ventricular assist device
LVEDD	Left ventricular end-diastolic diameter

## References

[1] Shah P., Yuzefpolskaya M., Hickey G.W., Breathett K., Wever-Pinzon O., Ton V.-K., Hiesinger W., Koehl D., Kirklin J.K., Cantor R.S. (2022). Twelfth Interagency Registry for Mechanically Assisted Circulatory Support report: Readmissions after left ventricular assist device. Ann. Thorac. Surg..

[2] Molina E.J., Shah P., Kiernan M.S., Cornwell W.K., Copeland H., Takeda K., Fernandez F.G., Badhwar V., Habib R.H., Jacobs J.P. (2021). The Society of Thoracic Surgeons Intermacs 2020 annual report. Ann. Thorac. Surg..

[3] Martin S.S., Aday A.W., Allen N.B., Almarzooq Z.I., Anderson C.A., Arora P., Avery C.L., Baker-Smith C.M., Bansal N., Beaton A.Z. (2025). 2025 Heart disease and stroke statistics: A report of US and global data from the American Heart Association. Circulation.

[4] Desai R.J., Mahesri M., Chin K., Levin R., Lahoz R., Studer R., Vaduganathan M., Patorno E. (2021). Epidemiologic characterization of heart failure with reduced or preserved ejection fraction populations identified using medicare claims. Am. J. Med..

[5] Rose S.W., Strackman B.W., Gilbert O.N., Lasser K.E., Paasche-Orlow M.K., Lin M., Saylor G., Hanchate A.D. (2024). Disparities by sex, race, and ethnicity in use of left ventricular assist devices and heart transplants among patients with heart failure with reduced ejection fraction. J. Am. Heart Assoc..

[6] Khazanie P. (2019). REVIVAL of the sex disparities debate: Are women denied, never referred, or ineligible for heart replacement therapies?. JACC Heart Fail..

[7] Joshi A.A., Lerman J.B., Sajja A.P., Dahiya G., Gokhale A.V., Dey A.K., Kyvernitakis A., Halbreiner M.S., Bailey S., Alpert C.M. (2019). Sex-based differences in left ventricular assist device utilization: Insights from the Nationwide Inpatient Sample 2004 to 2016. Circ. Heart Fail..

[8] Teuteberg J.J., Cleveland J.C., Cowger J., Higgins R.S., Goldstein D.J., Keebler M., Kirklin J.K., Myers S.L., Salerno C.T., Stehlik J. (2020). The Society of Thoracic Surgeons Intermacs 2019 annual report: The changing landscape of devices and indications. Ann. Thorac. Surg..

[9] Radhoe S.P., Jakus N., Veenis J.F., Timmermans P., Pouleur A., Rubís P., Van Craenenbroeck E.M., Gaizauskas E., Barge-Caballero E., Paolillo S. (2023). Sex-related differences in left ventricular assist device utilization and outcomes: Results from the PCHF-VAD registry. ESC Heart Fail..

[10] Gruen J., Caraballo C., Miller P.E., McCullough M., Mezzacappa C., Ravindra N., Mullan C.W., Reinhardt S.W., Mori M., Velazquez E. (2020). Sex differences in patients receiving left ventricular assist devices for end-stage heart failure. JACC Heart Fail..

[11] Maukel L.-M., Weidner G., Beyersmann J., Spaderna H. (2023). Adverse events after left ventricular assist device implantation linked to psychosocial risk in women and men. J. Heart Lung Transplant..

[12] Magnussen C., Bernhardt A.M., Ojeda F.M., Wagner F.M., Gummert J., de By T.M., Krabatsch T., Mohacsi P., Rybczynski M., Knappe D. (2018). Gender differences and outcomes in left ventricular assist device support: The European Registry for Patients with Mechanical Circulatory Support. J. Heart Lung Transplant..

[13] Maukel L.-M., Weidner G., Beyersmann J., Spaderna H. (2022). Sex differences in recovery and device replacement after left ventricular assist device implantation as destination therapy. J. Am. Heart Assoc..

[14] DeFilippis E.M., Breathett K., Donald E.M., Nakagawa S., Takeda K., Takayama H., Truby L.K., Sayer G., Colombo P.C., Yuzefpolskaya M. (2020). Psychosocial risk and its association with outcomes in continuous-flow left ventricular assist device patients. Circ. Heart Fail..

[15] Dew M.A., Hollenberger J.C.M., Obregon L.L.B., Hickey G.W., Sciortino C.M., Lockard K.L.M., Kunz N.M.R., Mathier M.A., Ramani R.N., Kilic A. (2021). The preimplantation psychosocial evaluation and prediction of clinical outcomes during mechanical circulatory support: What information is most prognostic?. Transplantation.

[16] Wang J., Okoh A.K., Chen Y., Steinberg R.S., Gangavelli A., Patel K.J., Ko Y.-A., Alexis J.D., Patel S.A., Vega D.J. (2024). Association of psychosocial risk factors with quality of life and readmissions 1 year after LVAD implantation. J. Card. Fail..

[17] Chew D.S., Manns B., Miller R.J.H., Sharma N., Exner D.V. (2017). Economic evaluation of left ventricular assist devices for patients with end stage heart failure who are ineligible for cardiac transplantation. Can. J. Cardiol..

[18] Baras Shreibati J., Goldhaber-Fiebert J.D., Banerjee D., Owens D.K., Hlatky M.A. (2017). Cost-effectiveness of left ventricular assist devices in ambulatory patients with advanced heart failure. JACC Heart Fail..

[19] Agrawal S., Garg L., Shah M., Agarwal M., Patel B., Singh A., Garg A., Jorde U.P., Kapur N.K. (2018). Thirty-day readmissions after left ventricular assist device implantation in the United States: Insights from the Nationwide Readmissions Database. Circ. Heart Fail..

[20] Imburgio S., Dandu S., Pannu V., Udongwo N., Johal A., Hossain M., Patel P., Sealove B., Almendral J., Heaton J. (2024). Sex-based differences in left ventricular assist device clinical outcomes. Catheter. Cardiovasc. Interv..

[21] Noly P.-E., Wu X., Hou H., Grady K.L., Stewart J.W., Hawkins R.B., Yang G., Kim K.D., Zhang M., Cabrera L. (2023). Association of days alive and out of the hospital after ventricular assist device implantation with adverse events and quality of life. JAMA Surg..

[22] Ahmed A., Adegbala O., Akintoye E., Inampudi C., Ajam M., Yassin A.S., Olawusi E., Shokr M., Alvarez P., Briasoulis A. (2020). Gender differences in outcomes after implantation of left ventricular assist devices. Ann. Thorac. Surg..

[23] Kirklin J.K., Pagani F.D., Kormos R.L., Stevenson L.W., Blume E.D., Myers S.L., Miller M.A., Baldwin J.T., Young J.B., Naftel D.C. (2017). Eighth annual INTERMACS report: Special focus on framing the impact of adverse events. J. Heart Lung Transplant..

[24] Bui Q.M., Allen L.A., LeMond L., Brambatti M., Adler E. (2019). Psychosocial evaluation of candidates for heart transplant and ventricular assist devices: Beyond the current consensus. Circ. Heart Fail..

[25] Dew M.A., DiMartini A.F., Dobbels F., Grady K.L., Jowsey-Gregoire S.G., Kaan A., Kendall K., Young Q.-R., Abbey S.E., Butt Z. (2018). Consensus Document: The 2018 ISHLT/APM/AST/ICCAC/STSW recommendations for the psychosocial evaluation of adult cardiothoracic transplant candidates and candidates for long-term mechanical circulatory support. J. Heart Lung Transplant..

[26] Ozga A.-K., Kieser M., Rauch G. (2018). A systematic comparison of recurrent event models for application to composite endpoints. BMC Med. Res. Methodol..

[27] Furberg J.K., Rasmussen S., Andersen P.K., Ravn H. (2022). Methodological challenges in the analysis of recurrent events for randomised controlled trials with application to cardiovascular events in LEADER. Pharm. Stat..

[28] van Buuren S., Groothuis-Oudshoorn K. (2011). Mice: Multivariate imputation by chained equations in R. J. Stat. Softw..

[29] Allignol A., Beyersmann J., Schmoor C. (2016). Statistical issues in the analysis of adverse events in time-to-event data. Pharm. Stat..

[30] Nießl A., Allignol A., Beyersmann J., Mueller C. (2023). Statistical inference for state occupation and transition probabilities in non-Markov multi-state models subject to both random left-truncation and right-censoring. Econom. Stat..

[31] Erdmann A., Beyersmann J., Bluhmki E. (2024). Comparison of nonparametric estimators of the expected number of recurrent events. Pharm. Stat..

[32] R Development Core Team (2018). R: A Language and Environment for Statistical Computing.

[33] Bauer G.R. (2023). Sex and gender multidimensionality in epidemiologic research. Am. J. Epidemiol..

[34] Schiebinger L., Leopold S.S., Miller V.M. (2016). Editorial policies for sex and gender analysis. Lancet.

[35] Heise L., Greene M.E., Opper N., Stavropoulou M., Harper C., Nascimento M., Zewdie D., Darmstadt G.L., Greene M.E., Hawkes S. (2019). Gender inequality and restrictive gender norms: Framing the challenges to health. Lancet.

[36] Regitz-Zagrosek V., Gebhard C. (2023). Gender medicine: Effects of sex and gender on cardiovascular disease manifestation and outcomes. Nat. Rev. Cardiol..

[37] Pelletier R., Khan N.A., Cox J., Daskalopoulou S.S., Eisenberg M.J., Bacon S.L., Lavoie K.L., Daskupta K., Rabi D., Humphries K.H. (2016). Sex versus gender-related characteristics: Which predicts outcome after acute coronary syndrome in the young?. J. Am. Coll. Cardiol..

[38] Regitz-Zagrosek V., Petrov G., Lehmkuhl E., Smits J.M., Babitsch B., Brunhuber C., Jurmann B., Stein J., Schubert C., Merz N.B. (2010). Heart transplantation in women with dilated cardiomyopathy. Transplantation.

[39] Steinberg R.S., Nayak A., Burke M.A., Aldridge M., Laskar S.R., Bhatt K., Sridharan L., Abdou M., Attia T., Smith A. (2022). Association of race and gender with primary caregiver relationships and eligibility for advanced heart failure therapies. Clin. Transplant..

[40] Breathett K., Yee E., Pool N., Hebdon M., Crist J.D., Yee R.H., Knapp S.M., Solola S., Luy L., Herrera-Theut K. (2020). Association of gender and race with allocation of advanced heart failure therapies. JAMA Netw. Open.

[41] Lee C.S., Sethares K.A., Thompson J.H., Faulkner K.M., Aarons E., Lyons K.S. (2020). Patterns of heart failure dyadic illness management: The important role of gender. J. Cardiovasc. Nurs..

[42] Connelly P.J., Azizi Z., Alipour P., Delles C., Pilote L., Raparelli V. (2021). The importance of gender to understand sex differences in cardiovascular disease. Can. J. Cardiol..

[43] Mwansa H., Lewsey S., Mazimba S., Breathett K. (2021). Racial/ethnic and gender disparities in heart failure with reduced ejection fraction. Curr. Heart Fail. Rep..

[44] Vogel B., Acevedo M., Appelman Y., Merz C.N.B., Chieffo A., A Figtree G., Guerrero M., Kunadian V., Lam C.S.P., Maas A.H.E.M. (2021). The Lancet women and cardiovascular disease Commission: Reducing the global burden by 2030. Lancet.

[45] Forest S.J., Xie R., Kirklin J.K., Cowger J., Xia Y., Dipchand A.I., Sivathasan C., Merry C., Lund L.H., Kormos R. (2018). Impact of body mass index on adverse events after implantation of left ventricular assist devices: An IMACS registry analysis. J. Heart Lung Transplant..

[46] Youmans Q.R., Zhou A., Harap R., Eskender M.H., Anderson A.S., Ezema A.U., Ghafourian K., Ohiomoba R., Pham D.T., Rich J.D. (2021). Association of cigarette smoking and adverse events in left ventricular assist device patients. Int. J. Artif. Organs.

[47] Havranek E.P., Mujahid M.S., Barr D.A., Blair I.V., Cohen M.S., Cruz-Flores S., Smith G.D., Himmelfarb C.D., Lauer M.S., Lockwood D.W. (2015). Social determinants of risk and outcomes for cardiovascular disease: A scientific statement from the American Heart Association. Circulation.

[48] Wever-Pinzon O., Drakos S.G., McKellar S.H., Horne B.D., Caine W.T., Kfoury A.G., Li D.Y., Fang J.C., Stehlik J., Selzman C.H. (2016). Cardiac recovery during long-term left ventricular assist device support. J. Am. Coll. Cardiol..

[49] McDonagh T.A., Metra M., Adamo M., Gardner R.S., Baumbach A., Böhm M., Burri H., Butler J., Čelutkienė J., Chioncel O. (2021). 2021 ESC Guidelines for the diagnosis and treatment of acute and chronic heart failure. Eur. Heart J..

[50] Heidenreich P.A., Bozkurt B., Aguilar D., Allen L.A., Byun J.J., Colvin M.M., Deswal A., Drazner M.H., Dunlay S.M., Evers L.R. (2022). 2022 ACC/AHA/HFSA guideline for the management of heart failure. Circulation.

[51] Nahlén Bose C. (2023). A meta-review of systematic reviews and meta-analyses on outcomes of psychosocial interventions in heart failure. Front. Psychiatry.

[52] Chernoff R.A.P., Messineo G.M., Kim S., Pizano D.M., Korouri S.B., Danovitch I.M., IsHak W.W.M. (2022). Psychosocial interventions for patients with heart failure and their impact on depression, anxiety, quality of life, morbidity, and mortality: A systematic review and meta-analysis. Psychosom. Med..

[53] Orth-Gomer K., Schneiderman N., Wang H.X., Walldin C., Blom M., Jernberg T. (2009). Stress reduction prolongs life in women with coronary disease: The stockholm women’s intervention trial for coronary disease (SWITCHD). Circ. Cardiovasc. Qual. Outcomes.

[54] Clancy M.J., Jessop A.B., Eisen H. (2019). Assessment of pre-operative psychosocial function among people receiving left ventricular assist devices: A national survey of US LVAD programs. Heart Lung.

[55] Bozkurt B., Savarese G., Eryd S.A., Bodegård J., Cleland J.G., Khordoc C., Kishi T., Thuresson M., Vardeny O., Zhang R. (2023). Mortality, outcomes, costs, and use of medicines following a first heart failure hospitalization: EVOLUTION HF. JACC Heart Fail..

[56] Vidula H., Takeda K., Estep J.D., Silvestry S.C., Milano C., Cleveland J.C., Goldstein D.J., Uriel N., Kormos R.L., Dirckx N. (2022). Hospitalization patterns and impact of a magnetically-levitated left ventricular assist device in the MOMENTUM 3 Trial. JACC Heart Fail..

[57] Shih H., Mondellini G.M., Kurlansky P.A., Sun J., Ning Y., Feldman V.R., Tiburcio M., Maguire C.W., Ladanyi A., Clerkin K. (2024). Unplanned hospital readmissions following HeartMate 3 implantation: Readmission rates, causes, and impact on survival. Artif. Organs.

