# Supplementary material for: Psychosocial Risk and Recurrent Hospitalizations in Women and Men Following LVAD Implantation: A Multi-State Analysis of the INTERMACS Registry
Source: J Cardiovasc Dev Dis. 2025 May 22;12(6):198. doi: 10.3390/jcdd12060198 (PMC12193544; doi:10.3390/jcdd12060198)
Supplement: Supplementary file 1 [file jcdd-12-00198-s001.zip › jcdd-3585012-supplementary.pdf]

Supplemental Material

Table S1. Reasons for rehospitalization in women and men with implanted left ventricular assist devices.

Reason for Rehospitalization	Women (%)	Men (%)
	n = 14018	n = 46851
Other, Specify	19.0	17.0
Major Infection	14.8	14.0
Major Bleeding	14.4	15.0
Neurological Dysfunction	5.6	5.2
Device Malfunction	4.8	4.6

Note. Five most prevalent reasons for rehospitalization by sex.

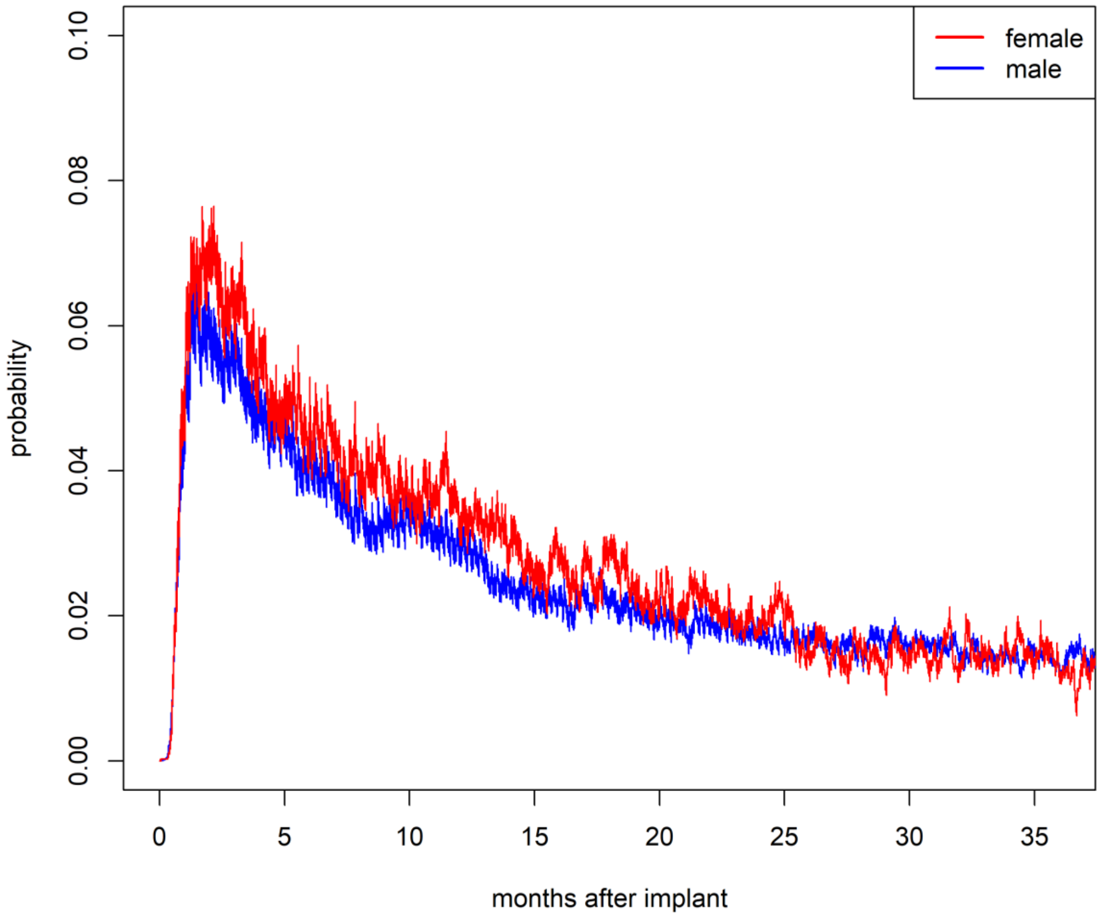

Figure S1. State occupation probabilities for being hospitalized stratified by sex.

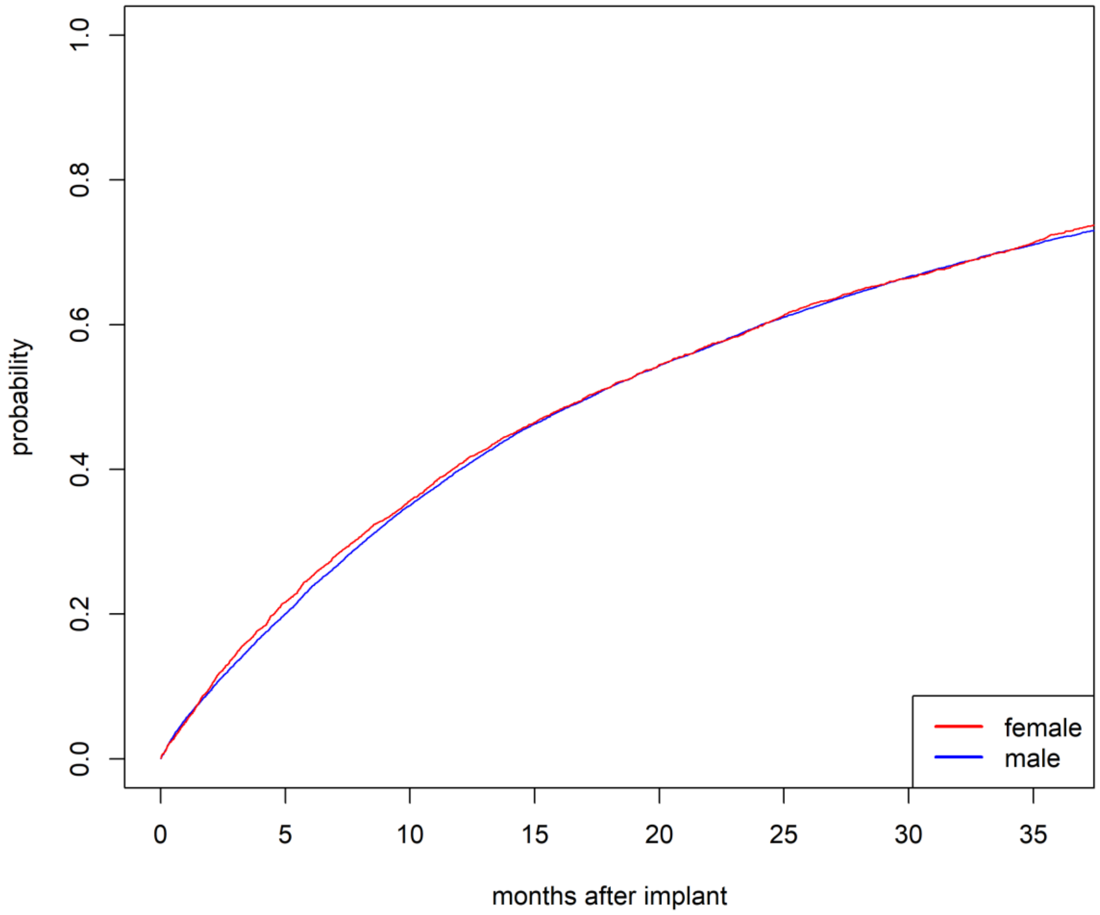

Figure S2. State occupation probabilities for being in any absorbing state (i.e., death, heart transplantation, device replacement due to complications, device explantation due to recovery) stratified by sex. In an absorbing state, patients are no longer at risk for subsequent events, such as rehospitalization.
